# Impact of systemic adjuvant therapy and CYP2D6 activity on mammographic density in a cohort of tamoxifen-treated breast cancer patients

**DOI:** 10.1007/s10549-021-06386-2

**Published:** 2021-09-27

**Authors:** Linda Thorén, Mikael Eriksson, Jonatan D. Lindh, Kamila Czene, Jonas Bergh, Erik Eliasson, Per Hall, Sara Margolin

**Affiliations:** 1 Department of Clinical Science and Education at Södersjukhuset, Karolinska Institutet, Stockholm, Sweden; 2 Department of Oncology, Södersjukhuset, Stockholm, Sweden; 3 Department of Medical Epidemiology and Biostatistics, Karolinska Institutet, Stockholm, Sweden; 4 Department of Laboratory Medicine, Clinical Pharmacology, Karolinska Institutet and Karolinska University Hospital, Stockholm, Sweden; 5 Department of Oncology-Pathology, Karolinska Institutet and Breast Cancer Center, Cancer Theme, Karolinska University Hospital, Karolinska Comprehensive Cancer Center, Stockholm, Sweden

**Keywords:** CYP2D6, Mammographic density, Tamoxifen, Adjuvant treatment, Breast cancer, Chemotherapy

## Abstract

**Purpose:**

Change in mammographic density has been suggested to be a proxy of tamoxifen response. We investigated the effect of additional adjuvant systemic therapy and CYP2D6 activity on MD change in a cohort of tamoxifen-treated pre- and postmenopausal breast cancer patients.

**Methods:**

Swedish breast cancer patients (*n* = 699)  operated 2006–2014, genotyped for CYP2D6, having at least three months postoperative tamoxifen treatment, a baseline, and at least one follow-up digital mammogram were included in the study. Other systemic adjuvant treatment included chemotherapy, goserelin, and aromatase inhibitors. Change in MD, dense area, was assessed using the automated STRATUS method. Patients were stratified on baseline characteristics, treatments, and CYP2D6 activity (poor, intermediate, extensive, and ultrarapid). Relative density change was calculated at year 1, 2, and 5 during follow-up in relation to treatments and CYP2D6 activity.

**Results:**

Mean relative DA decreased under the follow-up period, with a more pronounced MD reduction in premenopausal patients. No significant effect of chemotherapy, aromatase inhibitors, goserelin, or CYP2D6 activity on DA change was found. DA did not revert to baseline levels after tamoxifen discontinuation.

**Conclusion:**

Our results indicate that other systemic adjuvant therapy does not further reduce MD in tamoxifen-treated breast cancer patients. We could not confirm the previously suggested association between CYP2D6 activity and MD reduction in a clinical setting with multimodality adjuvant treatment. No rebound effect on MD decline after tamoxifen discontinuation was evident.

**Supplementary Information:**

The online version contains supplementary material available at 10.1007/s10549-021-06386-2.

## Introduction

Postoperative tamoxifen treatment for 5 years substantially reduces the risk for recurrence as well as breast cancer mortality. Women with high risk of recurrence are currently recommended extended treatment for 10 years [1], 2. Up to 40% of patients with early ER-positive breast cancer will, however, relapse within 20 years suggesting a wide variability in the response to adjuvant tamoxifen treatment [3]. Currently, there are no means of ascertaining the adjuvant effect of tamoxifen. Markers of early response to tamoxifen therapy are thus needed.

Tamoxifen, a selective estrogen-receptor modulator (SERM), inhibits estrogen-stimulated proliferation in ER-positive breast cancer by competitively binding to the ER [4]. Tamoxifen is a weak antiestrogen and requires hepatic biotransformation to more potent metabolites—most importantly endoxifen—by hepatic cytochrome P450 2D6 (CYP2D6) [5]. The CYP2D6 gene is highly polymorphic with more than 100 known allelic variants [6]. Each allele has been given an activity score (AS) ranging from 0–1. The sum of the AS values assigned to each allele are used to translate an individual´s CYP2D6 genotype into a phenotype [7].

Patients carrying a poor metabolizer [PM] genotype, approximately 5% of the European population [8], benefit less from tamoxifen treatment compared to intermediate [IM], extensive [EM], and ultrarapid [UM] metabolizers [9–12]. The clinical impact of CYP2D6 is, however, still controversial partly due to contradictory results from retrospective studies [13–15].

Change in mammographic density (MD) has been suggested as an early marker for tamoxifen response, indicating that women whose MD declined during tamoxifen therapy have a better prognosis than patients without a density decrease [16–23]. It has been hypothesized that the decline in MD may reflect levels of active tamoxifen metabolites. CYP2D6 status has been shown to influence the reduction in mammographic density during tamoxifen treatment in postmenopausal women [24].

Previous studies on breast density change have, however, mainly been conducted in patients receiving tamoxifen alone, as systemic treatment, a situation which is rarely seen in clinical practice, except in premenopausal patients with a low risk for recurrence. Patients at high risk of recurrence also generally receive anthracycline and/or taxane-based therapies, anti-Her2 treatments, and sometimes a switch between tamoxifen and aromatase inhibitors [25–27]. The knowledge of how systemic adjuvant therapy other than tamoxifen might affect MD change in breast cancer patients is limited [28]. The association between CYP2D6 activity and MD change in tamoxifen-treated breast cancer patients also receiving multimodality treatment is insufficiently studied [24].

Our aim was to investigate the effect of CYP2D6 activity and additional systemic treatment, including chemotherapy, GnRH-analogs and switch to aromatase inhibitors, on MD change, in a cohort of tamoxifen-treated pre- and postmenopausal breast cancer patients.

## Patients and methods

### Patients

Since 2006, DNA from peripheral blood has been collected and biobanked from newly diagnosed breast cancer patients at the Karolinska University Hospital and at Södersjukhuset, Stockholm, Sweden. From the national breast cancer registry (INCA), we identified 1256 breast cancer patients undergoing surgery January 2006–January 2014 in Stockholm. All of these patients had available DNA extracted from peripheral blood for CYP2D6 genotyping, were ER positive and initiated adjuvant tamoxifen treatment at the Departments of Oncology, Karolinska University Hospital or Södersjukhuset.

Inclusion criteria were having CYP2D6 genotyped, at least three months of adjuvant tamoxifen treatment according to patient records and a digital baseline mammogram followed by at least one digital follow-up mammogram. The baseline mammogram was defined as the latest available non-diagnostic mammogram prior to diagnosis of breast cancer. Date of follow-up mammogram should have been at least 3 months after diagnosis.

Patients were excluded if they did not receive tamoxifen upfront as adjuvant endocrine therapy, if they had been previously operated because of a contralateral breast cancer or were diagnosed with bilateral breast cancer at baseline. Patients who developed a contra lateral breast cancer or underwent contra lateral/ bilateral prophylactic mastectomy after study baseline contributed to the study up to the time of diagnosis or surgery. A detailed description of the selection of the study population is depicted in Online resource 1.

Information on menopausal status, tumor characteristics, body mass index (BMI), concomitant medication, systemic breast cancer treatment, compliance, side effects, and follow-up was obtained from medical records. Patients were considered postmenopausal one year after their last menstrual period or after bilateral oophorectomy. Patients with unknown menopausal status were defined as postmenopausal if age at diagnosis was >55 years. If there was unequivocal information in the record that the patient had stopped taking tamoxifen during the first five years, she was classified as non-compliant.

### Mammographic density measurement

Digital full-field mammograms from the mediolateral-oblique (MLO) breast view were retrieved up until January 2018. Mammographic dense area (DA), dense area/total breast area in cm^2^ of the left and right breasts, was measured using the fully automated STRATUS method. Before measurement and comparison were performed, images of the same breast were aligned to reduce technical differences between images, a method described previously [29]. The aligned density measurements from the image contralateral to the tumor from MLO view were averaged to form a single measurement for each examination. Mammographic density was measured at baseline and follow-up.

As we lacked information on BMI at follow-up, changes in the absolute DA rather than changes in PMD were chosen. PMD is highly, inversely correlated with BMI, whereas DA has been shown to be only weakly associated with BMI [30, 31].

### Genotyping of CYP2D6

The biobanked DNA was stored frozen at −80 °C and transported frozen for analysis. Analysis of CYP2D6 variant alleles was performed using TaqMan-based real-time PCR assays rigorously validated and implemented for routine clinical pharmacogenetic analyses at Diakonhjemmet Hospital in Oslo, Norway. The *CYP2D6* genotyping panel included the non-coding (“null”) variants CYP2D6 * 3 (*rs35742686*), CYP2D6 * 4 (*rs3892097*), CYP2D6 * 5 (gene deletion), and CYP2D6 * 6 (*rs5030655*), the decreased-function variants CYP2D6 * 9 (*rs5030656*), CYP2D6 * 10 (*rs1065852*), and CYP2D6 * 41 (*rs28371725*) and the increased-function variant CYP2D6 * 1XN. Without detection of any of the above variant alleles, the genotype was defined as CYP2D6 * 1/ * 1. In the copy number analysis, samples with gene CYP2D6 gene deletions were identified and interpreted as CYP2D6 * 5, whereas the allele multiplication/duplication analysis could not distinguish between CYP2D6 * 1, CYP2D6 * 4 and CYP2D6 * 41, leading to a simplified but commonly applied interpretation that the extra allele was fully active [9]. With regard to the latter limitation, an inconclusive genotyping result with regard to predicted activity (i.e., CYP2D6 * 1/ * 4, CYP2D6 * 1/ * 41 or CYP2D6 * 4/ * 41 in combination with CNA = 3 or 4) would lead to study exclusion.

### Predicted CYP2D6 activity

Each CYP2D6 allele was given an activity score (AS), according to The Clinical Pharmacogenetics Implementation Consortium (CPIC) guidelines. The sum of the AS values assigned to each allele was used to classify the patients into predicted CYP2D6 phenotypes; poor metabolizers, PM, (AS = 0), intermediate metabolizers, IM, (AS = 0.5 or 1.0), extensive metabolizers, EM, (AS = 1.5–2.0), or ultrarapid metabolizers, UM, (AS > 2.0) [32]. An alternative AS, categorizing patients with CYP2D6 *3/*4/*5/*6 in combination with CYP2D6 *9/*10/*41 as PM was also used [7, 33].

### Statistical analysis

Differences in characteristics of the included and not included women were estimated using Student’s t test on the continuous variables age, BMI, age at menopause, and treatment duration, and using Fisher’s exact test for the dichotomous variables menopausal status, use of tamoxifen and other endocrine treatment, chemotherapy, and CYP2D6 activity.

Baseline average mammographic density was analyzed using Local Polynomial Regression Fitting (LOESS) to investigate the trend across age [34].The fitted curve was plotted across age in relation to healthy women from the external KARMA cohort, including more than 70 000 women attending mammography screening between 2011 and 2013 in Sweden and from whom digital mammograms were stored [29]*.*

Average density decreases after initiation of tamoxifen treatment was plotted in pre- and postmenopausal women from the time of baseline until the end of follow-up, using Local Polynomial Regression Fitting (LOESS) with 95% confidence intervals. The study participants contributed with measures of mammographic density change till the end of follow-up or till time of medication discontinuation.

Relative density change was calculated for each follow-up mammogram as the follow-up density measurement minus the baseline density measurement, divided by the baseline density measurement [35]. Mean relative dense area decrease at year 1, 2, and 5 during follow-up was estimated using non-linear b-spline regression. Ninety-five percent confidence intervals were estimated using 1000 bootstrappings. Analyses were stratified by received treatments and CYP2D6 activities. The study participants contributed with measures of mammographic density change till the end of follow-up or till time of medication discontinuation. P-trends for CYP2D6 activities were estimated using linear regression in postmenopausal women at year 5.

Among women who discontinued tamoxifen, average density change was analyzed in the range 5 years prior to discontinuation until 5 years after discontinuation using non-linear b-spline regression [36]. The fitted model was plotted with 95% confidence intervals for pre- and postmenopausal women. Mammograms were performed yearly according to clinical routine during follow-up regardless whether the patient had discontinued the endocrine treatment or not. The mammograms closest to discontinuation and yearly mammograms thereafter were used in the analysis.

All statistical analyses were performed using SAS 9.4. All statistical tests were two sided, and statistical significance was set to an alpha 0.05.

## Results

In all, 699 women were included in the study. The majority (82%) of the patients received tamoxifen as their only adjuvant endocrine treatment during a mean treatment period of 4.1 years. Twelve percent of the patients switched from tamoxifen to an aromatase inhibitor, 6% were treated with a combination of tamoxifen and goserelin, and one percent received a combination of goserelin and aromatase inhibitor after tamoxifen. Notably, 26% of the patients had received neoadjuvant or adjuvant chemotherapy (56% of the premenopausal and 4% of the postmenopausal patients), consisting mostly of 6 courses of cyclophosphamide, epirubicin plus Fluorouracil (*5*-*FU*), with or without docetaxel. Five percent of the patients were treated with trastuzumab. Forty two percent of the patients in the study cohort were premenopausal.

Mean time between breast cancer diagnosis and first follow-up mammogram was 1.4 years. The corresponding figure between breast cancer diagnosis and last mammogram was 4.9 years (Table 1). Mammograms were available for 358 patients at year 1 of follow-up, 429 at year 2, 482 at year 3, 448 at year 4 and for 381 patients at year 5. 251 patients had available mammograms after more than 5 years of follow-up. According to information in medical records, 4% of the patients had, at some point during tamoxifen treatment, concomitant treatment with a clinically relevant CYP2D6 inhibitor. Four patients with undetermined CYP2D6 genotype were excluded. CYP2D6 metabolizer status was stratified according to CPIC guidelines to 7% PM, 36% IM, 54% EM, and 3% UM.

**Table 1:** Baseline characteristics of study participants and assigned treatments

Characteristics	Premenopausal	Postmenopausal	Whole cohort
*N* (%)	293 (58)	406 (42)	699 (100)
Age at breast cancer diagnosis mean, range, (SD)	46.2, 27–55 (4.9)	64.0, 47–82 (6.6)	56.5, 27–82 (10.6)
Body Mass Index (BMI) at baseline, kg/m^2^, mean, (SD)	25.2 (4.6)	26.1 (4.5)	25.7 (4.6)
Menopausal age, years, mean (SD)	–	50.0 (2.7)	50.0 (2.7)
Age at baseline mammogram, mean, years (SD)	46.0 (4.8)	63.8 (1.2)	56.3 (10.6)
Time from baseline mammogram to first follow-up mammogram, median, years (IQR)	1.5 (1.6)	1.4 (1.3)	1.4 (1.5)
Mammographic dense area (cm^2^) of fibro-glandular tissue, mean (SD)	47.9 (29.5)	26.8 (22.6)	35.5 (27.7)
Estrogen receptor positive, *N* (%)^a^	293 (100)	406 (100)	699 (100)
Progesterone receptor positive, *N* (%)^b^	290 (99)	402 (99)	692 (99)
Chemotherapy*, N* (%)	164 (56)	17 (4)	181 (26)
Endocrine treatment			
Tamoxifen only, *N* (%)	228 (78)	346 (85)	574 (82)
Tamoxifen and goserelin, *N* (%)	41 (14)	–	41 (6)
Tamoxifen and aromatase inhibitor, *N* (%)	24 (8)	60 (15)	84 (12)
Tamoxifen, goserelin, and aromatase inhibitor, *N* (%)	6 (2)	–	1
Tamoxifen treatment years, mean (SD)	4.3 (1.7)	4.0 (1.5)	4.1 (1.6)
CYP2D6 activity according to CPIC’s guidelines[32], *n* (%)			
CYP2D6 Poor Metabolizers (PM)	25 (8.5)	23 (5.7)	48 (6.9)
CYP2D6 Intermediate Metabolizers (IM)	107 (36.5)	222 (54.7)	252 (36.0)
CYP2D6 Extensive Metabolizers (EM)	153 (52.2)	145 (35.7)	375 (53.6)
CYP2D6 Ultrarapid Metabolizers (UM)	8 (2.7)	16 (3.9)	24 (3.4)
Follow-up time to last mammography, mean, years (SD)	4.9 (1.8)	4.9 (1.7)	4.9 (1.7)

Percentages may not add up to 100 due to rounding

Abbreviations: *SD* Standard Deviation, *IQR* Inter Quartile Range, *CYP2D6* Cytochrome P450 2D6, *CPIC* The Clinical Pharmacogenetics Implementation Consortium

^a^Tumors were considered Estrogen receptor (ER) positive and Progesterone receptor (PR) positive if ≥ 10% of the cells stained positive for the receptor by IHC on the resected tumor specimen and/ or pre-operative biopsy. 1 patient was ER negative, but PR positive and was, thus, defined as ER positive. For 3 patients, ER-status was missing. They were treated as ER positive

^b^For 7 patients, PR-status was missing

To identify a possible selection bias in our study sample, the excluded women from the full breast cancer patient population were compared with patients participating in the study. No significant differences were found regarding age, BMI, menopausal status, tumor size, ER/PR-status, Her2-status, positive lymph nodes, CYP2D6 status, or use of aromatase inhibitors. Patients included in the study had tumors with lower grade and proliferation and were more frequently treated with tamoxifen only as endocrine treatment, compared to patients not included in the study (82 vs 69%, *p* < 0.01). Study patients were concordantly less frequently treated with tamoxifen in combination with goserelin (6 vs 15%, *p* < 0.01) and had a longer treatment duration of tamoxifen (4.1 years, SD 1.6, vs 3.8 years SD2.0, *p* < 0.01) (Online resource 2).

**Fig. 1:**
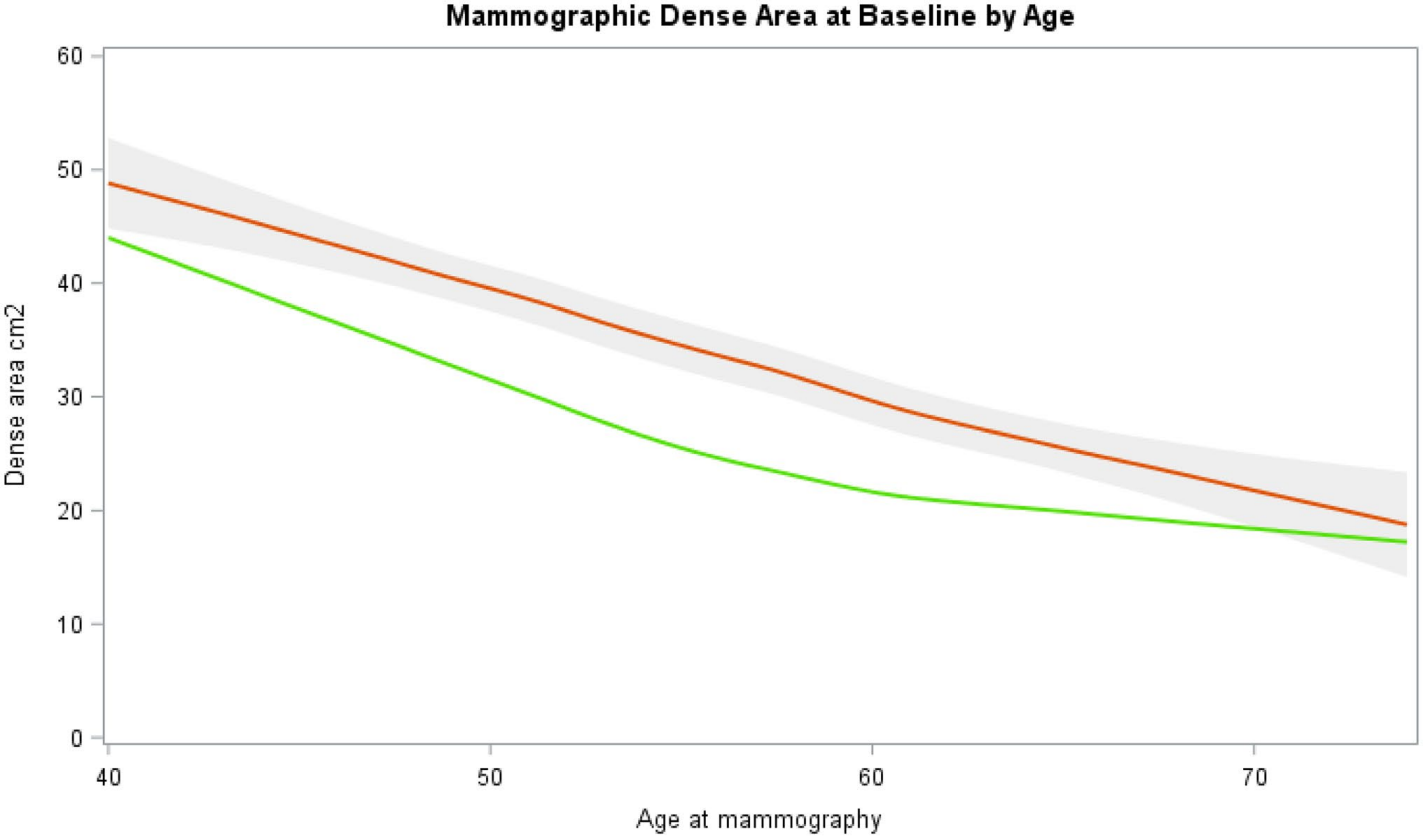
Mammographic dense area at baseline by age in breast cancer cases in tamoxifen cohort in relation to healthy women from the KARMA external cohort. Baseline average mammographic density was analyzed using Local Polynomial Regression Fitting (LOESS) to investigate the trend across age. The fitted curve was plotted across age in relation to healthy women from the external KARMA cohort (27). Red curve shows the breast cancer cases in the tamoxifen-treated cohort. Gray area shows the 95% confidence interval. Green curve shows average density across age in the same age range for women in the KARMA cohort

Mean absolute DA at baseline was 35.5 cm^2^ (SD 27.7). The average density at baseline was 50 cm^2^ among 40-year-old women and decreased to approximately 25 cm^2^ for women at the age of 74, with a clear decline during the menopausal transition (age 45–55). Mean DA at baseline was higher across age among study participants compared to healthy women in the external KARMA cohort (Fig. 1)*.*

**Fig. 2:**
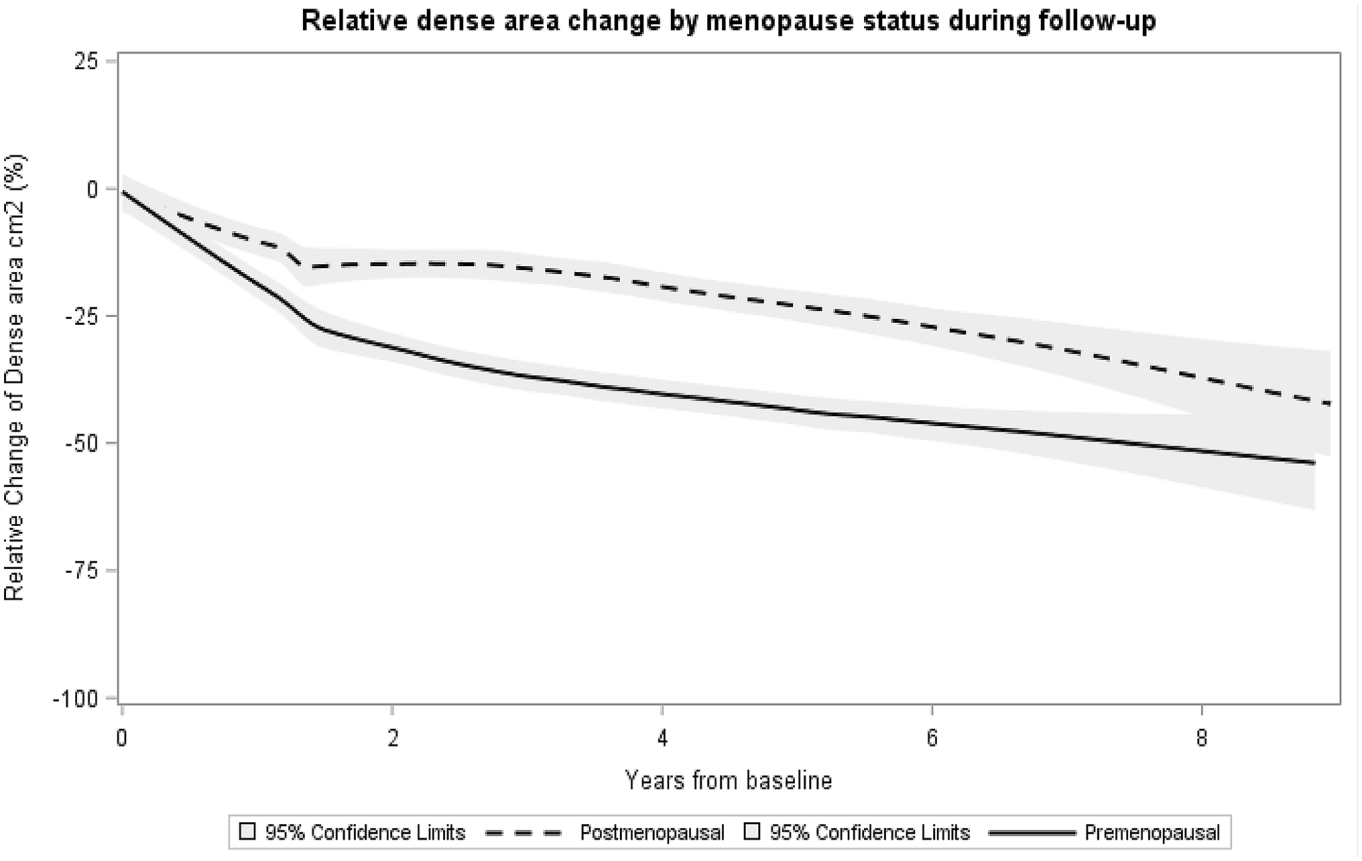
Relative density change of dense area at baseline until the end of follow-up in pre- and postmenopausal patients. Relative density change was calculated for each follow-up mammogram as the follow-up density measurement minus the baseline density measurement, in turn divided by the baseline density measurement. Average density decrease after initiation of tamoxifen treatment was plotted in pre- and postmenopausal women from the time of baseline until the end of follow-up, using Local Polynomial Regression Fitting (LOESS). Ninety-five percent confidence intervals were estimated using boot strapping. The study participants contributed with measures of mammographic density change till the end of follow-up or till time of medication discontinuation

During the first year, DA change was more pronounced in the premenopausal group compared to the postmenopausal women (Fig. 2), a difference that disappeared beyond one year after diagnosis.

Mean relative density change at year one of follow-up was −18.1 (CI-22.9, −13.9) in the whole cohort, −22.2 (CI-26.6, −16.6) and −15.9 (CI-22.7, −9.0) in the pre- and postmenopausal subgroups, respectively. In the patients with tamoxifen monotherapy, the corresponding mean relative density change was −18.2 (CI-23.2, −14.0) in the whole group, −23.0 (CI-26.2, −19.0) among the premenopausal patients and −15.0 (CI-22.2, −7.4) in the postmenopausal cohort. As expected, the relative density change increased over time. The density decrease in the chemotherapy-treated premenopausal group was similar to patients treated with tamoxifen only. Density decrease had similar effect estimates in chemo-treated pre- and postmenopausal patients at year 1 and 2. In the postmenopausal chemotherapy-treated subgroup, consisting of 17 patients, a MD decrease was, however, seen compared to postmenopausal women treated with tamoxifen only.

No additional effect of aromatase inhibitors, goserelin, or CYP2D6 activity on DA decrease was observed. We did observe what appears to be a trend of density reduction with higher CYP2D6 activity scores at year 5 among postmenopausal patients who received tamoxifen treatment only and for all patients who also received chemotherapy, *p* = 0.05 (Table 2), (Online resource 3).

**Table 2:** Relative density change (%) at year 1, 2, and 5 during follow-up in relation to received treatments and CYP2D6 activity

	Premenopausal^a^ (CI), *n*	Postmenopausal^a^ (CI), *n*	Combined (CI), *n*
Treatment			
* Year 1*			
All tamoxifen-treated women	−22.2 (−26.6, −16.6), n = 143	−15.9 (−22.7, −9.0), n = 215	−18.1(−22.9, −13.9),n = 358
* Tamoxifen only subgroup*^b^	−23.0 (−26.2, –19.0), *n* = 114	−15.0 (−22.2, −7.4),*n* = 184	−18.2 (−23.2, −14.0), *n* = 298
* Chemotherapy subgroup*	−22.9 (−28.8, −14.8), *n* = 81	−21.3 (−42.0, 7.7), *n* = 9	−22.6 (−28.3, −14.7), n = 90
* Goserelin subgroup*	−15.8 (−29.4, 1.7), *n* = 17	N/A	−15.8 (−29.4, 1.7), *n* = 17
* Aromatase inhibitor subgroup*	−25.3 (−40.7, −12.2), *n* = 14	−14.2 (−34.9, −0.9), *n* = 31	−19.0 (−31.9, −8.7), *n* = 45
* Year 2*			
All tamoxifen-treated women	−31.8 (−36.2, −27.8), *n* = 170	−13.1 (−17.5, −7.6), *n* = 259	−21.1(−24.4, −16.6), *n* = 429
* Tamoxifen only subgroup*^b^	−32.7(−37.3, −27.6) *n* = 138	−14.1 (−18.3, −8.5), *n* = 223	−21.5 (−24.8, −17.4), *n* = 361
* Chemotherapy subgroup*	−35.1 (−41.8, −29.3), *n* = 97	−35.1 (−58.5, −18.9), *n* = 11	−35.8 (−42.7, −29.8),n = 108
* Goserelin subgroup*	−27.7 (−39.8, −16.6), *n* = 16	N/A	−27.7 (−39.8, −16.6), *n* = 16
* Aromatase inhibitor subgroup*	−30.9 (−46.1, −19.5), *n* = 19	−10.9 (−21.3, 3.2), *n* = 36	−19.1 (−27.0, −9.8), n = 55
* Year 5*			
All tamoxifen-treated women	−43.6 (−47.7, −38.5), *n* = 162	−24.8 (−31.0, −19.7), *n* = 219	−32.4 (−36.8, −28.7), *n* = 381
* Tamoxifen only subgroup*^b^	−44.5 (−48.9, −39.5), *n* = 127	−24.2 (−29.7, −19.6), *n* = 185	−32.2 (−37.0, −28.6), *n* = 312
* Chemotherapy subgroup*	−44.6 (−51.1, −33.8), *n* = 94	−35.8 (−61.8, −17.4), *n* = 12	−43.2 (−49.3, −33.9), *n* = 106
* Goserelin subgroup*	−47.4(−59.7, −32.9), *n* = 18	N/A	−47.4(−59.7, −32.9), *n* = 18
* Aromatase inhibitor subgroup*	−36.2 (−49.7, −9.8), *n* = 18	−25.3 (−38.9, −12.0), *n* = 34	−28.2 (−37.9, −17.0), *n* = 52
CYP2D6 activity^c^			
* Year 1*			
PM	−24.2 (−38.9, −11.2), *n* = 10	−5.6 (−27.2, 18.4), *n* = 14	−15.2 (−28.4, −2.9), *n* = 24
IM	−14.3 (−22.7, −4.0), *n* = 55	−14.4 (−29.5, −3.7), *n* = 69	−13.8 (−21.1, −8.2), *n* = 124
EM	−26.0 (−34.9, −19.2), *n* = 75	−17.2 (−25.6, −9.8), *n* = 123	−21.3(−28.6, −15.4), *n* = 198
UM	−24.0 (−50.6, 8.5), *n* = 3	2.1 (−45.1, 58.5), *n* = 9	−2.2 (−35.4, 120.8), *n* = 12
* Year 2*			
PM	−35.0 (−46.7, −25.2), *n* = 12	−11.1 (−25.6, 0.9), *n* = 16	−23.1 (−33.5, −15.2), *n* = 28
IM	−30.7 (−39.8, −22.8), *n* = 60	−9.43 (−16.7, 0.5), *n* = 84	−18.7 (−23.8, −12.9), *n* = 144
EM	−32.6 (−37.6, −27.2), *n* = 94	−16.3 (−21.4, −9.7), *n* = 149	−22.5 (−26.7, −17.1), *n* = 243
UM	−39.9 (−65.6, 1.4), *n* = 4	−11.4 (−41.6, 14.7), *n* = 10	−19.9 (−42.7, 2.9), *n* = 14
* Year 5*			
PM	−52.9 (−63.1, −36.2), *n* = 11	−15.0 (−28.9, 5.5), *n* = 11	−33.7(−43.0, −13.5), *n* = 22
IM	−44.1 (−53.2, −35.9), *n* = 64	−21.2 (−30.5, −13.1), *n* = 78	−30.9 (−39.1, −25.2), *n* = 142
EM	−42.4 (−47.7, −33.5), *n* = 81	−26.4 (−34.9, −20.4), *n* = 120	−32.6(−38.1, −27.7), *n* = 201
UM	−51.3 (−73.9, −31.1), *n* = 6	−39.3 (−61.5, −20.2), *n* = 10	−43.0 (−57.8, −29.0), *n* = 16
CYP2D6 activity^c^ in tamoxifen only subgroup			
* Year 1*			
PM	−25.5 (−37.8, −11.6), *n* = 7	−1.4 (−24.0, 60.3), *n* = 12	−13.4 (−25.8, 2.0), *n* = 19
IM	−16.1 (−24.4, −6.1), *n* = 44	−16.5 (−33.3, −4.9), *n* = 57	−15.2 (−23.8, −9.5), *n* = 101
EM	−27.4 (−36.8, −20.7), *n* = 60	−16.4 (−25.2, −8.7), *n* = 109	−20.9 (−27.8, −14.8), *n* = 169
UM	−19.9 (−58.2, 13.2), *n* = 3	−6.4 (−104.0, 72.8), *n* = 6	−11.0 (−67.8, 39.9), *n* = 9
* Year 2 *			
PM	−40.0 (−51.3, −29.1), *n* = 9	−12.1 (−28.5,4.6), *n* = 13	−24.7 (−36.3, −13.6), *n* = 22
IM	−29.2 (−39.7, −21.4), *n* = 52	−7.6 (−15.1, 2.8), *n* = 67	−17.7 (−22.7, −10.8), *n* = 119
EM	−34.4 (−40.4, −27.5), *n* = 74	−17.4 (−23.6, −11.4), *n* = 134	−23.5 (−28.0, −18.4), *n* = 208
UM	−33.6 (−59.7, 19.5) *n* = 3	−14.5 (−44.7, 16.3), *n* = 9	−19.6 (−44.6, 5.5), *n* = 12
* Year 5*			
PM	−59.4 (−69.4, −45.8), *n* = 6	−16.0 (−32.4, 8.2), *n* = 9	−35.6 (−48.0, −16.1), *n* = 15
IM	−42.4 (−54.4, −33.6), *n* = 50	−18.9 (−28.0, −9.5), *n* = 62	−28.4 (−36.0, −22.5), *n* = 112
EM	−44.6 (−51.3, −37.9), *n* = 65	−27.1 (−36.0, −20.1), *n* = 105	−33.5 (−39.3, −28.4), *n* = 170
UM	−50.6 (−71.7, −29.4), *n* = 6	−42.6 (−65.8, −23.0), *n* = 9	−45.6 (−62.9, −31.1), *n* = 15

Mean relative dense area decrease was estimated using non-linear b-spline regression. Ninety-five percent confidence intervals were estimated using boot strapping. The study participants contributed with measures of mammographic density change till the end of follow-up or till time of medication discontinuation. P-trends for CYP2D6 activities were estimated using linear regression in postmenopausal women at year 5. The p-trend was 0.05 for the patients who received tamoxifen treatment only and 0.05 for all patients who also received chemotherapy. *N/A* Not applicable

Abbreviations: *CI* Confidence interval, *CYP2D6* Cytochrome P450 2D6, *CPIC* The Clinical Pharmacogenetics Implementation Consortium

^a^Menopause status at study baseline

^b^Subgroup with tamoxifen only and w/o chemotherapy, goserelin, aromatase inhibitor treatment

^C^A CYP2D6 activity score (AS) according to CPIC was used to classify patients into predicted phenotypes: poor metabolizers (PM), intermediate metabolizers (IM), extensive metabolizers (EM), or ultrarapid metabolizers (UM) [32]

**Fig. 3:**
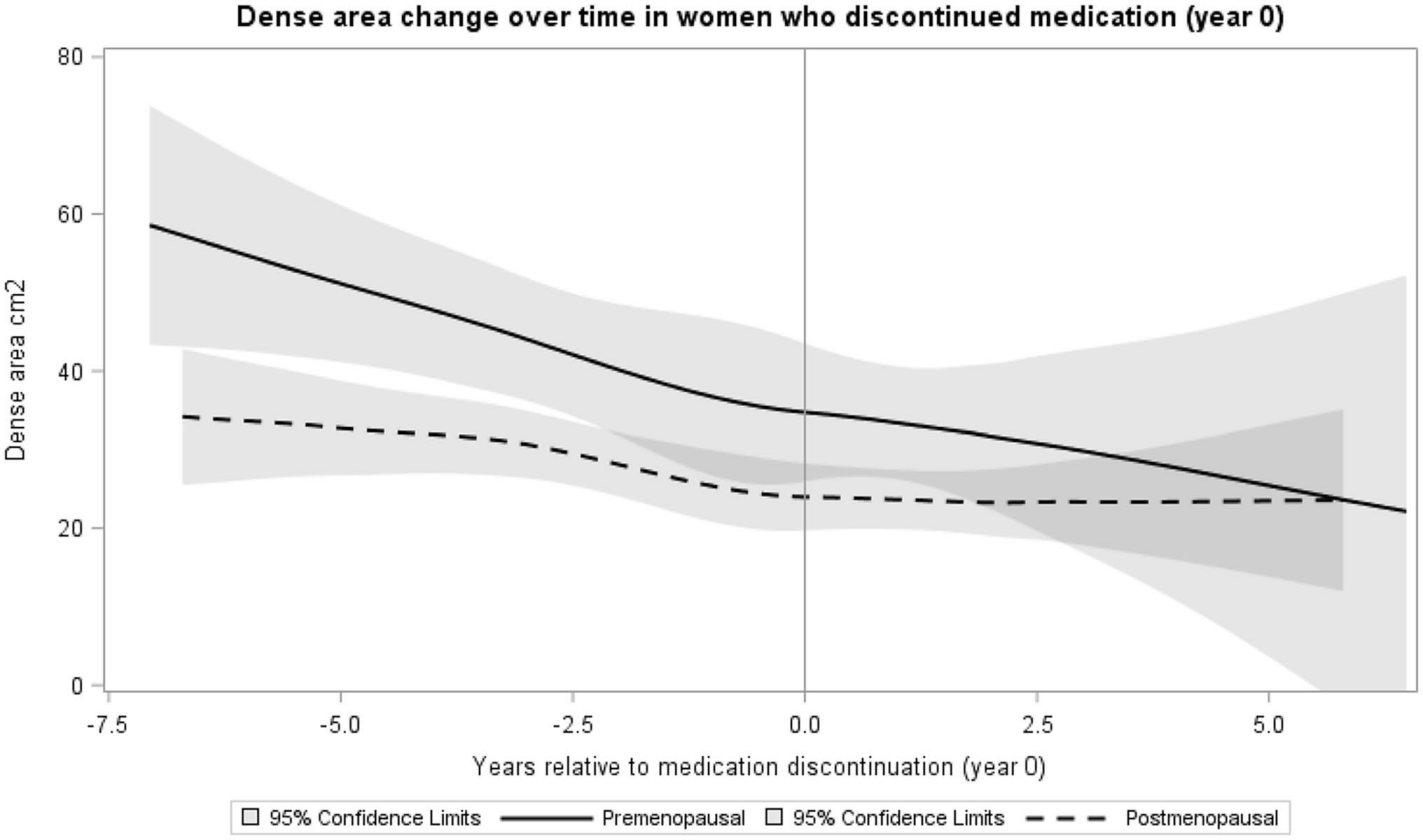
Dense area change over time in patients who discontinued tamoxifen. Among women who discontinued tamoxifen, average density change was estimated for women who had their closest available mammograms between 5 years prior to discontinuation and 5 years after discontinuation (n=89 patients). Time zero is the time of discontinuation. Time scale is given in years between the density measurement and tamoxifen discontinuation using discontinuation as the reference (year 0). The density estimates were based on non-linear b-spline regression over the 10-year interval in premenopausal (N=29) and postmenopausal (N=60) women. The fitted model was plotted with 95% confidence intervals for pre- and postmenopausal women. The premenopausal women were 51.9 (SD 4.8) years at tamoxifen discontinuation with 4.9 (SD 2.6) years of follow-up. The postmenopausal women were 67.4 years at tamoxifen discontinuation (SD 6.8) and had 3.9 years of follow-up (SD 1.8)

In all, 192 patients (27%) discontinued tamoxifen within 5 years of treatment start. No rebound in breast density after tamoxifen discontinuation was evident in the 29 of the premenopausal women and 60 of the postmenopausal women for whom mammograms after tamoxifen cessation were available (Fig. 3)*.* The premenopausal women were on average 51.9 (SD 4.8) years at tamoxifen discontinuation and had 4.9 (SD 2.6) years of follow-up. The mean age for the postmenopausal women at tamoxifen discontinuation was 67.4 years (SD 6.8) and their mean follow-up was 3.9 years (SD 1.8). No further density decrease was seen in women discontinuing tamoxifen and switching to aromatase inhibitors or goserelin (data not shown).

## Discussion

In this cohort of tamoxifen-treated breast cancer patients neither aromatase inhibitors, goserelin nor chemotherapy were associated with significant additional mammographic density change. A previously reported effect of CYP2D6 activity on density decrease was not possible to confirm in the present study. Furthermore, stopping tamoxifen early did not, in our material, lead to a subsequent increase to baseline MD levels.

Higher breast density is a strong risk factor for breast cancer [20, 37], and MD at baseline was indeed higher among our study participants than in healthy women compared by age (Fig. 1) [29]. The baseline density was comparable to the breast cancer cohort by Nyante et al. with a similar age span [38]. The decline in mammographic density was greatest during the menopausal transition, supporting an underlying hormonal mechanism for breast density change.

Several previous studies, including a recent prevention trial [35], have shown promising results using reduction in mammographic density as an early marker of adjuvant tamoxifen response. This could be important additional information when prescribing and choosing long-term adjuvant endocrine therapy, with substantial side effects, to breast cancer patients. These studies have, however, mainly been conducted in patient cohorts treated with tamoxifen alone, as systemic treatment. In the modern clinical reality, many patients receive multimodality treatment.

Most studies evaluating density change associated with adjuvant tamoxifen have used mammograms obtained around 12 to 18 months after cancer diagnosis [17]. MD assessment has, however, differed by mammogram modality and methodology (analog vs. digital mammograms, absolute vs. relative MD change and in the age span of included patients), which makes comparisons difficult. For instance, a study by Li et al. 2013 observed that more than 50% of the patients experienced a MD decrease of at least 20% at their 12- to 18-month mammogram [21]. In line with previous studies, the degree of MD reduction under tamoxifen treatment was more pronounced in the premenopausal group [39], 22 (Fig. 2). Chemotherapy frequently induces menopause in premenopausal patients and may, thus, be an important confounder for MD decrease in this group. Only a few previous studies have taken this into account. Previous data suggest that chemotherapy treatment reduces MD, especially in younger women. As these studies have not been designed to analyze the additional impact of chemotherapy on MD in tamoxifen-treated patients, it is difficult to compare our results with previous data [18, 28, 40, 41]*.* In this study, no significant additional effect of chemotherapy on MD decrease in premenopausal women was evident. The postmenopausal group treated with chemotherapy consisted of only 17 patients and importantly, nearly 50% (8/17) had been diagnosed with breast cancer within a year after menopause, suggesting a not completely extinguished ovarian function as a likely explanation for the pronounced density decrease observed in this subgroup.

Only one previous study has investigated the impact of goserelin on MD decrease in tamoxifen-treated breast cancer patients [42]. In contrast to this small report we did not find an association between ovarian suppression and MD decrease. The number of patients in our study treated with goserelin was, however, limited and whether tamoxifen-induced density reduction might be enhanced by ovarian suppression needs to be further studied in a larger cohort. In line with previous data, no effect on density was found in patients changing their endocrine treatment from tamoxifen to aromatase inhibitors [22, 28, 43].

Compliance to tamoxifen is an important clinical concern. A Swedish register study found that 31% of patients prescribed adjuvant endocrine treatment were non-adherent after three years [44], another that 50% discontinued their endocrine treatment before five years treatment [45]. In our study, 7% of the patients discontinued tamoxifen within the first year and 27% before completing 5 years of treatment. Only a few studies have investigated tamoxifen adherence and MD change and results have been inconsistent [16], 19, 28.

Interestingly, we did not detect an increase in MD after stopping tamoxifen, neither in the pre- nor in the postmenopausal patients. Although the number of patients for whom data on MD change after tamoxifen discontinuation was limited, our results suggest that in women with a MD reduction, MD does not revert to baseline levels after tamoxifen intake ends. The MD decline seemed to plateau for the postmenopausal patients after tamoxifen discontinuation, and we cannot conclude that the MD decline would have continued if tamoxifen therapy had not stopped. Age-related declines may also occur upon tamoxifen discontinuation, especially in the premenopausal patients, as they approached menopause. The majority of the premenopausal patients at diagnosis were likely close to menopause at the time of tamoxifen discontinuation. Further assessment of MD change after tamoxifen treatment, especially in younger women is warranted.

No effect of predicted CYP2D6 activity on density decrease could be confirmed in this study. We did observe what appears to be a borderline significant trend of density reduction with higher CYP2D6 activity scores among postmenopausal women who received tamoxifen only and for all women who also received chemotherapy. Our results indicate that CYP2D6 activity does not substantially modify MD response in patients receiving multimodality adjuvant treatment. An association between CYP2D6 genotype and mammographic density change under tamoxifen treatment might be more difficult to prove in patients receiving complex adjuvant therapy. Larger sample sizes might be needed. The biological basis of the tamoxifen-dependent reduction of MD is not yet fully clarified and other factors than CYP2D6 status are also likely of importance.

Strengths of this study include the prospective collection of consecutive unselected breast cancer cases and detailed clinical data generated from individual medical records including additional adjuvant treatment and compliance to tamoxifen. CYP2D6 was extensively genotyped on DNA from blood. By using only digital images and the STRATUS system, measurement variability in MD change was reduced [29]. One limitation is our study cohort size, where 44% of the patients of the original full cohort had to be excluded, mainly due to the unavailability of digital mammograms. Digitalization of mammograms in Stockholm was initiated in 2006 but not complete until 2010, which explains the majority of missing images. No significant differences were, however, found between the study base and the full cohort regarding factors most relevant to mammographic density change. Other limitations include incomplete information on concomitant medication with CYP2D6 inhibitors and information on adherence from medical records only. Therefore, the true figure for adherence to tamoxifen might be lower. Although an association between CYP2D6 genotype and mammographic density change under tamoxifen treatment might be more difficult to demonstrate in patients receiving complex adjuvant therapy, it is altogether a strength that our study reflects modern clinical practice.

In summary, in our cohort of tamoxifen-treated breast cancer patients, other systemic adjuvant therapy does not appear to further reduce MD. Moreover, no rebound effect on MD decline after early tamoxifen discontinuation was evident. An effect of CYP2D6 activity on density decrease was not possible to confirm in the present material, with patients receiving multimodality adjuvant treatment. Future studies with larger cohorts and with consideration of adherence are warranted. More work is also needed to clarify whether critical plasma concentrations of active tamoxifen metabolites are required for MD decline under tamoxifen treatment and whether patients with no MD change on tamoxifen would benefit from switching endocrine therapy.

## Supplementary Information

Below is the link to the electronic supplementary material.

Supplementary file1 (PDF 88 kb)

Supplementary file2 (DOC 48 kb)

Supplementary file3 (PDF 57 kb)

## Data Availability

The data that support the findings of this study are available from the corresponding author upon reasonable request.

## Code availability

The code that support the analyses of this study are available from the corresponding author upon reasonable request.
