# Supplementary figures and images for: Impact of systemic adjuvant therapy and CYP2D6 activity on mammographic density in a cohort of tamoxifen-treated breast cancer patients
Source: Breast Cancer Res Treat. 2021 Sep 27;190(3):451–62. doi: 10.1007/s10549-021-06386-2 (PMC8558195; doi:10.1007/s10549-021-06386-2)

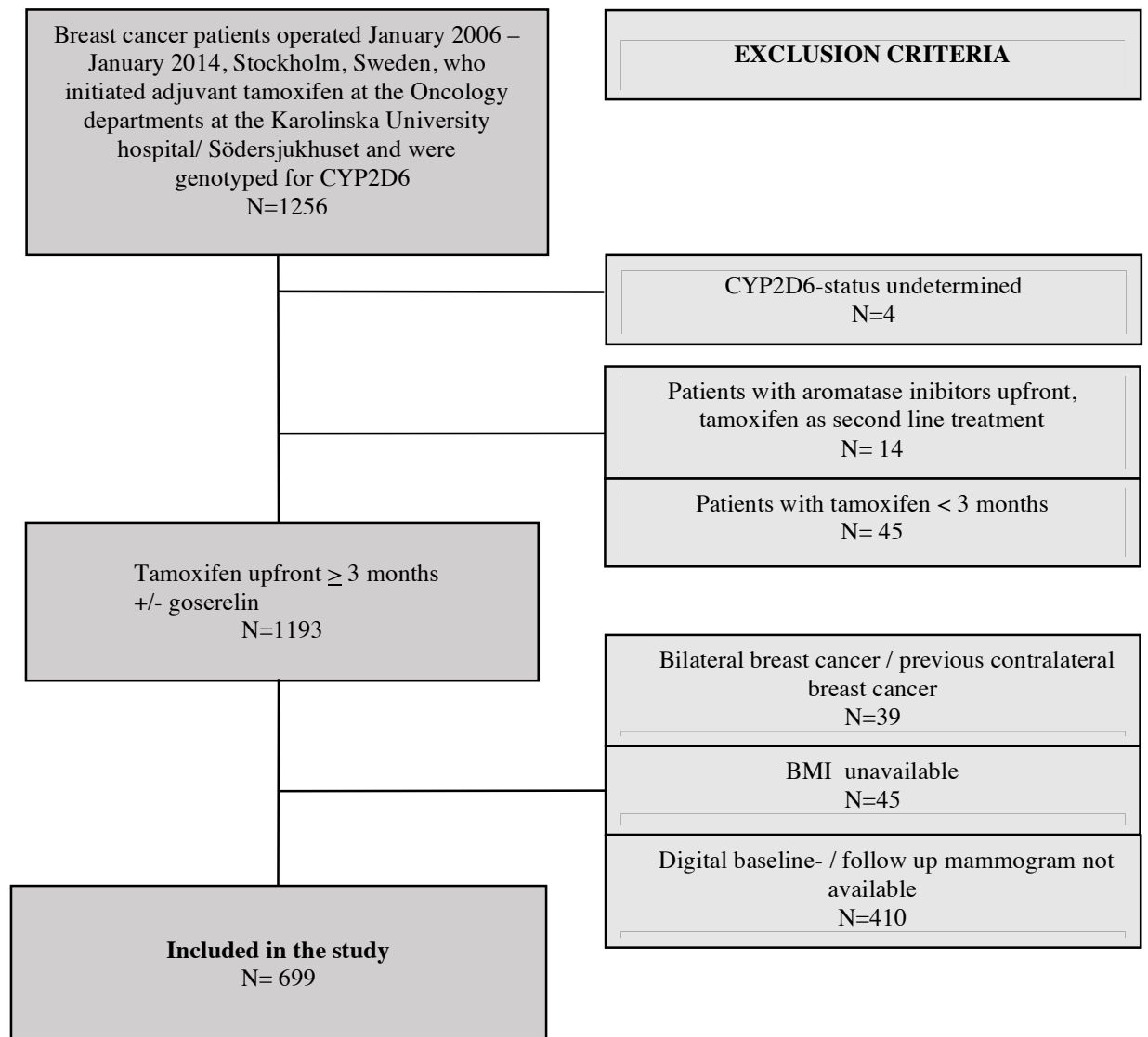

#### Online resource 1

Inclusion and exclusion criteria for study participants

Supplement: Supplementary file 1 — Supplementary file1 (PDF 88 kb) [file 10549_2021_6386_MOESM1_ESM.pdf]
