# Supplementary material for: Impact of systemic adjuvant therapy and CYP2D6 activity on mammographic density in a cohort of tamoxifen-treated breast cancer patients
Source: Breast Cancer Res Treat. 2021 Sep 27;190(3):451–62. doi: 10.1007/s10549-021-06386-2 (PMC8558195; doi:10.1007/s10549-021-06386-2)
Supplement: Supplementary file 2 — Supplementary file2 (DOC 48 kb) [file 10549_2021_6386_MOESM2_ESM.doc]

**Online Resource 2**

Study population after exclusion criteria in relation to full genotyped population.

| **Characteristics** | **Included in study** | **Not included in study** | **p value** |
| --- | --- | --- | --- |
| N | 699 | 557 | - |
| Age at breast cancer diagnosis, range (SD) | 56.5, (10.6) | 57.2, (14.3) | 0.29 |
| Body Mass Index (BMI) at baseline, kg/m^2^, mean (SD) | 25.7 (4.8) | 25.5 (5.2) | 0.45 |
| Postmenopausal, %  Menopausal age, years, mean (SD) | 58  50.0 (2.7) | 60  49.9 (2.7) | 0.42  0.49 |
| Tumor size:  <=20 mm, %  21mm - <=50 mm, %  > 50mm, %  Estrogen-receptor positive, %  Progesterone-receptor positive, %  Her2-positive, %  Grade according to Elston-Ellis;  1  2  3  Postitive Lymph nodes, %  S phase > 10% / Ki _67_ > 20, % | 73  21  6  100  88  4.5  37  50  14  18  22 | 69  25  5  99  83  5.6  29  51  20  22  29 | 0.20  0.99  0.05  0.36  <0.01  0.07  <0.01 |
| Chemotherapy*,%* | 26 | 29 | 0.28 |
| Endocrine treatment  Tamoxifen only, %  Tamoxifen and goserelin, %  Tamoxifen and aromatase inhibitor, %  Tamoxifen, goserelin and aromatase inhibitor, % | 82  6  12  0.9 | 69  15  14  1.4 | <0.01  <0.01  0.40  0.42 |
| Tamoxifen treatment years, (mean, SD) | 4.1 (1.6) | 3.8 (2.0) | <0.01 |
| CYP2D6- activity according to CPIC’s guidelines [32], n (%)  CYP2D6 Poor Metabolizers (PM)  CYP2D6 Intermediate Metabolizers (IM)  CYP2D6 Extensive Metabolizers (EM)  CYP2D6 Ultrarapid Metabolizers (UM) | 48 (6.9)  252 (36.0)  375 (53.6)  24 (3.4) | 43 (7.8)  196 (35.4)  296 (53.5)  18 (3.2) | 0.80 |

Abbreviations:

SD: Standard Deviation, Her2: human epidermal growth factor receptor 2, CYP2D6: Cytochrome P450 2D6, CPIC: The Clinical Pharmacogenetics Implementation Consortium.

The study base was compared with the group of patients from the full cohort that were not included, using Student’s t-test on continuous variables; age, BMI, age at menopause and treatment duration. To evaluate differences in menopausal status, use of tamoxifen/ other endocrine treatment, and chemotherapy between the study base and patients from the full cohort that were not included, Fisher´s exact test was used. CYP2D6-activity and tumor size were evaluated using the Wilcoxon test.
