# Supplementary material for: Impact of systemic adjuvant therapy and CYP2D6 activity on mammographic density in a cohort of tamoxifen-treated breast cancer patients
Source: Breast Cancer Res Treat. 2021 Sep 27;190(3):451–62. doi: 10.1007/s10549-021-06386-2 (PMC8558195; doi:10.1007/s10549-021-06386-2)
Supplement: Supplementary file 3 — Supplementary file3 (PDF 57 kb) [file 10549_2021_6386_MOESM3_ESM.pdf]

Online resourse 3. Odds ratios of density decrease by 10 percent or more between follow up and baseline at any time during study follow up in relation to CYP2D6 activity.

CYP2D6 activity	Premenopausal[a] (CI)	Postmenopausal (CI)	Combined (CI)
**All included patients,**			
**Linear CYP2D6 activity,**			
**- AS according to CPIC[b]**	0.91 (0.61-1.37)	1.12 (0.83-1.51)	1.01 (0.79-1.30)
**- Alternative AS[c]**	0.98 (0.67-1.43)	1.05 (0.81-1.38)	1.02 (0.81-1.28)
**All included patients,**			
**Categorized CYP2D6-activity,**			
**- AS according to CPIC[b]**			
IM vs PM	0.41 (0.11-1.48)	1.32 (0.54-3.21)	0.73 (0.34-1.60)
EM vs PM	0.50 (0.14-1.79)	1.53 (0.64-3.64)	0.87 (0.41-1.89)
UM vs PM	0.40 (0.05-3.00)	1.16 (0.32-4.19)	0.68(0.21-2.25)
**- Alternative AS[c]**			
IM vs PM	0.78 (0.30-2.03)	0.83 (0.41-1.70)	0.81 (0.44-1.46)
EM vs PM	0.89 (0.36-2.22)	1.05 (0.53-2.05)	0.96 (0.55-1.70)
UM vs PM	0.71 (0.12-4.31)	0.80 (0.25-2.54)	0.75 (0.26-2.20)
**Patients with tamoxifen only,**			
**Linear CYP2D6 activity,**			
**- AS according to CPIC[b]**	0.86 (0.52-1.40)	1.28 (0.92-1.77)	1.05 (0.78-1.40)
**- Alternative AS[c]**	0.90 (0.57-1.42)	1.17 (0.87-1.56)	1.02 (0.78-1.34)
**Patients with tamoxifen only,**			
**Categorized CYP2D6-activity,**			
**- AS according to CPIC[b]**			
IM vs PM	0.20 (0.03-1.58)	1.30 (0.50-3.39)	0.51 (0.16-1.59)
EM vs PM	0.27 (0.03-2.11)	1.67 (0.66-4.26)	0.67 (0.21-2.07)
UM vs PM	0.15 (0.01-1.97)	2.00 (0.48-8.36)	0.54 (0.12-2.39)
**- Alternative AS[c]**			
IM vs PM	0.45 (0.12-1.68)	0.77 (0.36-1.17)	0.59 (0.27-1.26)
EM vs PM	0.60 (0.16-2.17)	1.10 (0.53-2.27)	0.81 (0.39-1.69)
UM vs PM	0.33 (0.04-2.50)	1.32 (0.36-4.85)	0.66 (0.20-2.20)
**Patients with tamoxifen only,**			
**no chemotherapy,**			
**Linear CYP2D6 activity,**			
**- AS according to CPIC[b]**	1.09 (0.56-2.12)	1.33 (0.95-1.86)	1.20 (0.83-1.75)
**- Alternative AS[c]**	1.20 (0.65-2.21)	1.21 (0.89-1.63)	1.20 (0.86-1.70)
**Patients with tamoxifen only,**			
**no chemotherapy,**			
**Categorized CYP2D6-activity,**			
**- AS according to CPIC[b]**			
IM vs PM	0.42 (0.05-3.82)	1.55 (0.56-4.29)	0.80 (0.24-2.72)
EM vs PM	0.87 (0.09-8.04)	1.98 (0.73-5.38)	1.31 (0.39-4.44)
UM vs PM	0.17 (0.01-3.05)	2.50 (0.58-10.88)	0.65 (0.13-3.29)
**- Alternative AS[c]**			
IM vs PM	1.03 (0.28-4.67)	0.85 (0.38-1.89)	0.94 (0.40-2.20)
EM vs PM	0.94 (0.44-8.60)	1.20 (0.57-2.54)	1.53 (0.66-3.51)
UM vs PM	0.38 (0.04-4.04)	1.52 (0.41-5.65)	0.76 (0.20-2.94)

Abbreviations:
CI: confidence interval, CYP2D6: Cytochrome P450 2D6, CPIC: The Clinical Pharmacogenetics Implementation Consortium.

a) Menopause status at study baseline.
b) A CYP2D6 activity score (AS) according to CPIC was used to classify patients into predicted phenotypes; poor metabolizers, PM, (AS = 0), intermediate metabolizers, IM, (AS = 0.5 or 1.0), extensive metabolizers, EM, (AS = 1.5 - 2.0), or ultrarapid metabolizers, UM, (AS > 2.0) [32].

c) An alternative AS, categorizing patients with CYP2D6 *3/*4/*5/*6 in combination with CYP2D6 *9/*10/*41 as PM was used [33,7].

The factors included in the table were estimated using logistic regression comparing density responders with non-responders after stratification by menopausal status and adjustment for age. Density response was defined based on mammographic density measures of all available mammograms during the follow-up period compared with the mammographic density measurement of the baseline mammogram.
